# Chitosan nanoparticles as a promising tool in nanomedicine with particular emphasis on oncological treatment

**DOI:** 10.1186/s12935-021-02025-4

**Published:** 2021-06-24

**Authors:** Javad Sharifi-Rad, Cristina Quispe, Monica Butnariu, Lia Sanda Rotariu, Oksana Sytar, Simona Sestito, Simona Rapposelli, Muhammad Akram, Mehwish Iqbal, Akash Krishna, Nanjangud Venkatesh Anil Kumar, Susana S. Braga, Susana M. Cardoso, Karolina Jafernik, Halina Ekiert, Natália Cruz-Martins, Agnieszka Szopa, Marcelo Villagran, Lorena Mardones, Miquel Martorell, Anca Oana Docea, Daniela Calina

**Affiliations:** 1 Phytochemistry Research Center, Shahid Beheshti University of Medical Sciences, Tehran, Iran; 2 Facultad de Medicina, Universidad del Azuay, Cuenca, Ecuador; 3 Facultad de Ciencias de La Salud, Universidad Arturo Prat, Avda. Arturo Prat 2120, 1110939 Iquique, Chile; 4 Banat’s University of Agricultural Sciences and Veterinary Medicine “King Michael I of Romania” From Timisoara, Calea Aradului 119, 300645 Timis, Romania; 5 Department of Plant Biology Department, Institute of Biology, Taras Shevchenko National University of Kyiv, Kyiv, 01033 Ukraine; 6 Department of Plant Physiology, Slovak University of Agriculture, Nitra, 94976 Slovak Republic; 7 Department of Pharmacy, University of Pisa, Via bonanno 6, 56126 Pisa, Italy; 8 Department of Eastern Medicine and Surgery, Directorate of Medical Sciences, GC University Faisalabad, Faisalabad, Pakistan; 9 Institute of Health Management, Dow University of Health Sciences, Karachi, Pakistan; 10 Department of Chemistry, Manipal Institute of Technology, Manipal Academy of Higher Education, Manipal, 576104 India; 11 LAQV-REQUIMTE, Department of Chemistry, University of Aveiro, 3810-193 Aveiro, Portugal; 12 Department of Pharmaceutical Botany, Medical College, Jagiellonian University, Medyczna 9, 30-688 Kraków, Poland; 13 Faculty of Medicine, University of Porto, Porto, Portugal; 14 Institute for Research and Innovation in Health (i3S), University of Porto, Porto, Portugal; 15 Institute of Research and Advanced Training in Health Sciences and Technologies, Cooperativa de Ensino Superior Politécnico e Universitário (CESPU), 4585-116 Gandra, Portugal; 16 Biomedical Science Research Laboratory and Scientific-Technological Center for the Sustainable Development of the Coastline, Universidad Catolica de La Santisima Concepcion, Concepcion, Chile; 17 Department of Nutrition and Dietetics, Faculty of Pharmacy, and Centre for Healthy Living, University of Concepción, 4070386 Concepción, Chile; 18 Department of Toxicology, University of Medicine and Pharmacy of Craiova, 200349 Craiova, Romania; 19 Department of Clinical Pharmacy, University of Medicine and Pharmacy of Craiova, 200349 Craiova, Romania

**Keywords:** Cancer, Chitosan, Nanomedicine, Targeted therapy, Nanoparticles

## Abstract

The study describes the current state of knowledge on nanotechnology and its utilization in medicine. The focus in this manuscript was on the properties, usage safety, and potentially valuable applications of chitosan-based nanomaterials. Chitosan nanoparticles have high importance in nanomedicine, biomedical engineering, discovery and development of new drugs. The manuscript reviewed the new studies regarding the use of chitosan-based nanoparticles for creating new release systems with improved bioavailability, increased specificity and sensitivity, and reduced pharmacological toxicity of drugs. Nowadays, effective cancer treatment is a global problem, and recent advances in nanomedicine are of great importance. Special attention was put on the application of chitosan nanoparticles in developing new system for anticancer drug delivery. Pre-clinical and clinical studies support the use of chitosan-based nanoparticles in nanomedicine. This manuscript overviews the last progresses regarding the utilization, stability, and bioavailability of drug nanoencapsulation with chitosan and their safety.

## Cancer as a global problem and recent advances in nano-delivery for cancer treatment

A malignant tumour is an unusual state in which a cluster of cells ignore the normal functional rules of the cell distribution, and develop in an uncontrolled way [1]. Malignant cells don’t react to the signs that stimulate the normal cell cycle as they have a degree of self-adequacy, which leads to the uncontrolled growth and production of altered cells [38]. If the multiplication of cancerous cells persists, it can be lethal. 90% of fatalities associated with cancers are a consequence of the spread of cancer cells to other tissues, known also as metastasis [2]. Cancer is one of the principal causes of death globally, with an expected 7.6 million persons lost their lives every year and accounting for 13% of all demises. Mortality related to cancer is anticipated to increase to 13.1 million by the year 2030 [3].

Cancer is not only an illness, but it includes a huge number of diseases with every organ or system growing a different set of ailments [4, 5]. About 30% of cancer associated death are related to smoking, additional lifestyle factors [6], or dietetic practices. To some extends, several types of cancers are avoidable by modifying unhealthy lifestyle habits. [7, 39–41].

One of the prospective essential advantages of nanotechnology for the treatment of carcinoma is tumour targeting [8]. Europe reports 23.4% of the carcinoma cases worldwide and 20.3% of the carcinoma fatalities, though it has only 9.0% of the global population. The United States of America have 13.3% of the international populace and account for 2% of occurrence and 14.4% of cancer related deaths globally. In comparison with other regions of the world, the ratios of deaths caused by metastasis cancer in Africa and Asia (7.3% and 57.3% respectively) are more than the percentages of incident cases (5.8% and 48.4% correspondingly), since these areas have a raised incidence of certain types of cancer-related with the worst prognosis and elevated rates of death, in addition to restricted access to well-timed identification and management.

Some of the cancers, such as lung cancer, breast cancer in females, and colorectal carcinomas, are the top 3 types of cancers in terms of frequency. They are categorized among the top 5 in terms of death (first, fifth, and second, respectively). Collectively, these 3 types of cancer are accountable for 1/3^rd^ of the cancer prevalence and death burden worldwide. Carcinomas of the lungs and breast are the most important types globally in expressions of the quantities of novel cases; for each of these types, around 2.1 million case detections are approximated in 2018, contributing about 11.6% of the overall cancer frequency burden. Colorectal carcinoma (1.8 million cases) is the 3rd most frequently identified cancer [9], carcinoma of the prostate is the 4th (1.3 million cases), and gastric carcinoma is the 5^th^ (1.0 million cases, 5.7%). Pulmonary carcinoma is the most frequently identified cancer in males, i.e., 14.5% of the overall cases in males and 8.4% in females along with the principal root of death by cancer in males 22%, which is about 1 in five of all cancer mortalities. In men, this is chased by CA prostate 13.5% and colorectal carcinoma 10.9% for frequency and liver carcinoma 10.2% and gastric carcinoma 9.5% for death. Carcinoma of the breast is the most frequently identified cancer in women [10] and cancer is the most widespread in 154 out of the one hundred and eighty-five countries included in GLOBOCAN 2018 [42]. Carcinoma of the breast is also the principal cause of cancer demise in women i.e., 15%, followed by pulmonary carcinoma 13.8% and colorectal carcinoma 9.5%, which are also the 3^rd^ and 2^nd^ most widespread types of cancer, correspondingly; CA Cervix ranks 4^th^ for both frequency (6.6%) and death (7.5%) [43]. Statistics show that there is a continuing need to make progress in target drug delivery systems in cancer therapy because oral administration of anticancer drugs is extremely difficult due to physiological barriers in the gastrointestinal tract such as poor solubility and low permeability of the intestinal membrane.

A good example is Docetaxel, a powerful anticarcinogenic medicine used in the management of prostate cancer, breast carcinoma, non-small-cell pulmonary carcinoma, and stomach adenocarcinomas. Docetaxel is considered one of the leading anticancer medicines in clinical utilization [44]. Studies showed that the highest concentration of drugs in plasma obtained after nanoparticle form administration was fourfold higher than that of the Docetaxel solution. Moreover, the highest concentration of plasma was achieved afterwards for the nanoparticle formulation contrasted to the free drug, proposing a persistent-release profile. The prolonged-releasing property of the nanoparticles amplified the distribution time of the drug, so the encapsulation of drugs into the nanoparticles might have also a protective role against drug degradation [45]. Docetaxel nanoparticles considerably restrained the growth of the tumour. Studies showed that the nanocrystals accomplished maximum effectiveness of medicine loading, with a small number of toxic effects in contrast with the presently commercially accessible Cremophor-filled formulations following oral experience [46].

The capacity to distinguish carcinogenic cells from non-carcinogenic and to discriminatingly eliminate malignant cells is essential for the aim of nanotechnology. In the case of the oral Paclitaxel administration, studies showed that the concentrations of the drug in plasma were two times higher for the nanoparticles compared with the commercially available Paclitaxel [47].

## Chitosan: from chemical properties to perspective of pharmaceutical uses

Chitosan is the denomination given to a range of polymers obtained from chitin, a natural polysaccharide composed of β-(1,4)-linked N-acetyl glucosamine units [19, 20]. The most common sources of chitin include fungi and the exoskeleton of crustaceans and insects.

**Fig. 1:**
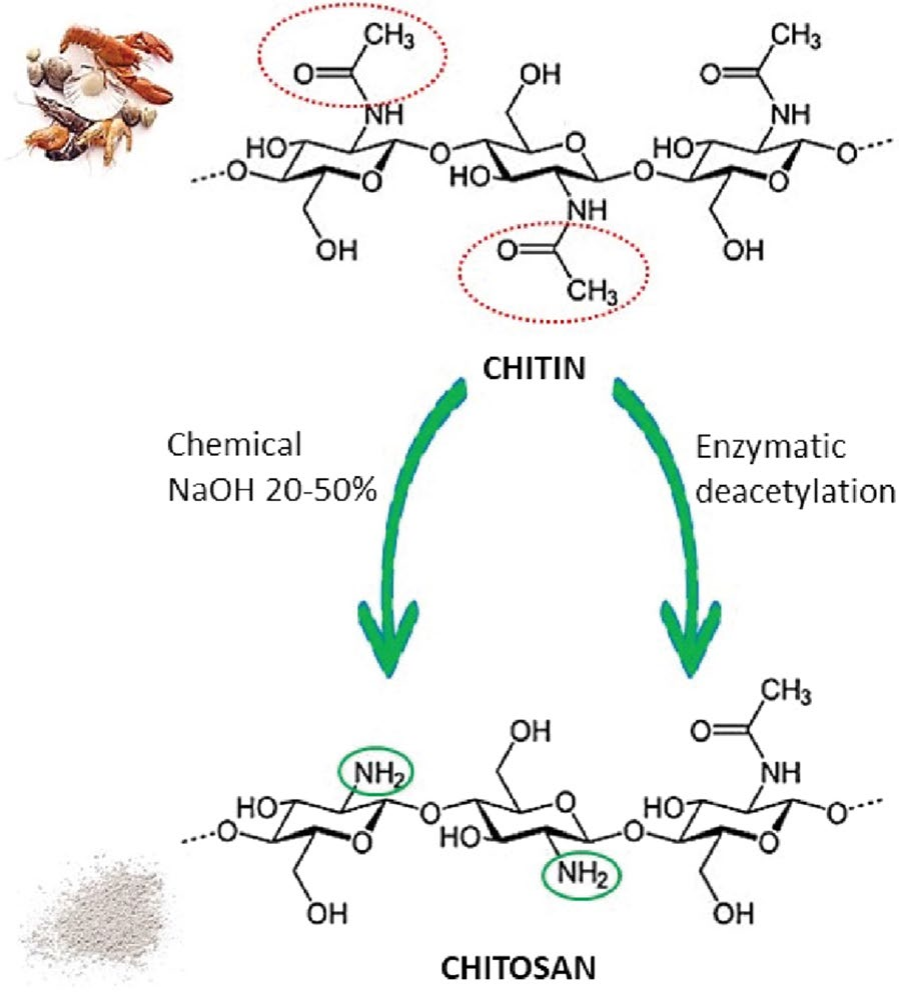
Summarized scheme of chitin deacetylation into chitosan

The transformation of chitin into chitosan is achieved by deacetylation. The process can be either chemical, using a strong solution of sodium hydroxide (25–50%) and high temperature (90–120 ºC), or biochemical, using deacetylases. (Fig. 1).

According to the conditions used in the deacetylation reaction, the resulting chitosan polymers will have different lengths, and also different remaining acetyl residues. This translates into a large range of molecular weights, from 300 to over 1000 kD. Moreover, the degree of acetylation of chitosan, ranging from 5 to 70% [21], has a strong influence on physicochemical properties such as viscosity and solubility [22]. Chitosan, while being insoluble in water at neutral pH, can dissolve in dilute acids owing to the protonation of its free amine groups. Cationic chitosan is thus soluble in dilute acetic, formic, citric, and other acids [23], in direct correlation to its deacetylation degree.

The amine groups of chitosan also influence a large variety of its pharmaceutical and biomedical properties, including mucoadhesion, permeation enhancement, transfection, and in situ gelation [24, 25]. Adding to the broad range of bioactive features, chitosan benefits from good biotolerability, low immunogenicity, and facile biodegradation in vivo [25].

The pharmaceutical use of chitosan requires a careful choice of the material. Given the wide diversity of chitosan polymers available, choosing the right molecular weight, degree of acetylation, and purity grade may appear to be a troublesome task. Some guidelines can be found in the European (6^th^ edition) and United States (29^th^ edition) Pharmacopoeias [26, 27], namely regarding purity and degree of acetylation, albeit the later has a rather broad tolerance interval (Table 1). The most frequent impurities present in chitosan are ash, heavy metals, and proteins. Proteins are relevant to biological activity because they can bring immunogenicity issues. High ash and residual protein content may hamper dissolution and cause difficulties in the preparation of chitosan-based drug delivery systems [28].

**Table 1:** Acceptance criteria for chitosan and chitosan hydrochloride according to the European and US Pharmacopoeias [26, 27]

Parameter	Eur. Ph. 6.0 Chitosan·HCl	USP Ph. 34-NF 29 Chitosan
Appearance	White solid	–
Degree of deacetylation	70.0%–95.0%	70.0%–95.0%
Distribution of molecular weight^a^	–	0.85–1.15
pH of 1% (g/mL) solution	4.0–6.0	–
Loss on drying^a^	–	< 5%
Total Impurities/Insolubles	≤ 0.5%	≤ 1.0%
Heavy metals	≤ 40 ppm	≤ 10 ppm
Iron	–	≤ 10 ppm
Sulfated ash^a^	≤ 1.0%	–
Protein	–	≤ 0.2%

^a^Determined on a sample weighing 1.0 g

The stability of chitosan is also a very important factor to consider when aiming at pharmaceutical applications. Chitosan is very sensitive to environmental conditions, especially humidity, due to its high hygroscopy. Water retention on chitosan occurs by hydrogen bonding, and it was reported to change its mechanical properties [29] and cause a partial loss of its mucoadhesive properties [30]. Thermal degradation of chitosan solutions was also observed, both at ambient temperature and at 60ºC [31, 32]. For these reasons, storage at low temperatures (2–8 ºC) in a dry ambient is recommended.

Chitosan-based nanoparticles can be used for the delivery of active ingredients, such as drugs or natural products, by diverse routes of administration such as oral and parenteral delivery [33, 34]. Chitosan nanoparticles are also particularly suitable for local delivery at the dermis and the mucosa, namely in the nasal, buccal, pulmonary and rectal routes, owing to the mucoadhesive properties and permeability-enhancing action of chitosan [33–35]. Chitosan nanoparticles combine the natural properties of the polymer with tuneable size and the possibility of surface modification according to custom needs, being thus a very promising and versatile strategy to overcome the bioavailability and stability issues of most active ingredients [33].

Nowadays, chitosan nanoparticles have become of great interest in nanomedicine, biomedical engineering, and the development of new therapeutic drug release systems with improved bioavailability increased specificity and sensitivity, and reduced pharmacological toxicity. Some activities may depend on the form and size of chitosan nanoparticles.

The novel chitosan nanoparticles composed of clusters of nanoparticles with sizes ranging from 10 to 80 nm shown potential for nanomedicine, biomedical engineering, industrial, and pharmaceutical fields [36]. The chitosan-based nanosystems can be used as advanced drug delivery systems in large part due to their remarkable physicochemical and biological characteristics due to their capacity to alter protein loading and adjust the value of each parameter during preparation. They also have high stability, high protein packing efficiency can be prepared as a lyophilized powder, and are easy to store and transport [37].

## Nanotechnology and its utilization

Nanotechnology is described as the study and utilization of structures between 1 to 100 nm in size. Nanotechnology is the synergy of chemical engineering, mechanical, microelectronics, electrical, material sciences, and biological screening. Nanotechnologies are the production, plan, categorization, and application of devices, structures, and systems by managing shape and size at the nanometer scale [11]. There are already more than three hundred declared products of nanotechnology in the market [48].

Nanoparticles can be described as particles less than 100 nm in diameter that demonstrates novel or improved size-dependent properties contrasted with bigger particles of similar material. Nanoparticles subsist broadly in the natural world [49]. Nanotechnology proposes immense visions of developed, individualized management of ailment.

The expectation is that individualized medicine will make it likely to develop and administer the suitable drug, at the proper dose, at the right time to the right patient. The advantages of this approach are safety, accuracy, speed, and efficacy. At this time, the most progressive part of nanomedicine is the development and utilization of nanoparticles for the delivery of drugs.

**Fig. 2:**
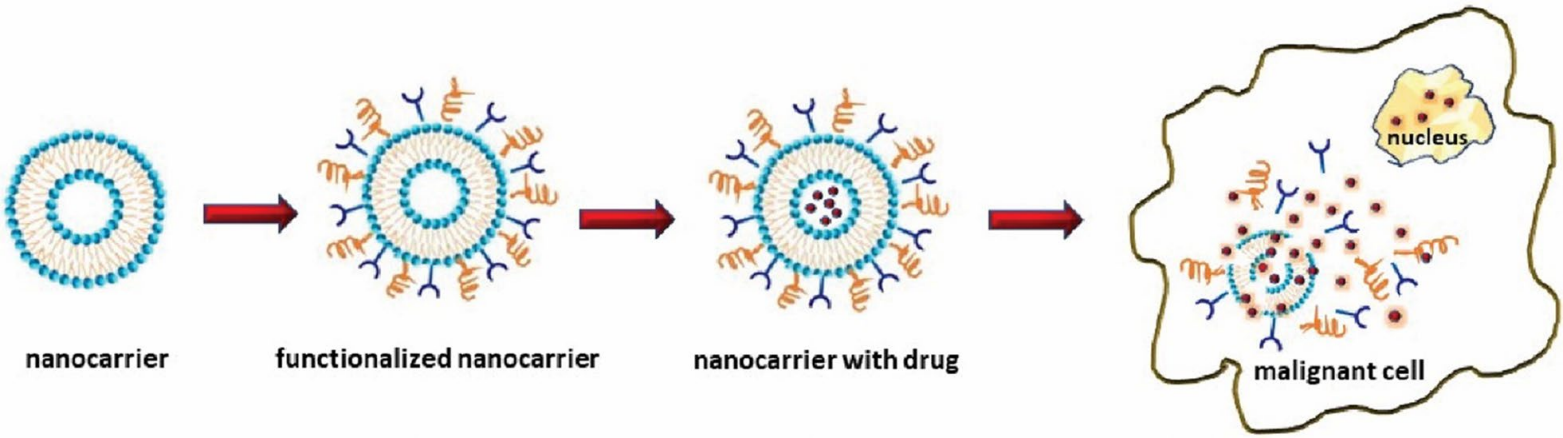
Nanoparticles (liposomes) used for the target transport of anticancer drugs

Nanomedicines have become well esteemed in modern times due to nanostructures utilization as delivery agents by encapsulating drugs and targeted delivery in specific tissues [50, 51]. Nanoparticle- based products have been developed both for imaging in cancer diagnosis and also for pharmacotherapeutic management [52]. The first generation of nanoparticles-based products comprised of lipid systems like micelles and liposomes, which were approved for food and drug manufacturing [53]. (Fig. 2).

These micelles and liposomes can have inorganic nanoparticles like magnetic or gold Np [53]. Nano compositions persist in the blood circulatory system for an extended period and facilitate the prolonged release of the carried drug. These specific releases regiments decrease the drug concentration fluctuation in plasma leading to a decrease of side effects.

Regarding the utilization of nanomaterials in drug delivery, the selection of the Np is based on the physicochemical characteristics of medicines [12]. The combined utilization of nanoscience in conjunction with bioactive natural compounds is very appealing and emerging quite rapidly nowadays. It presents several benefits when is used for the delivery of natural products for the treatment of several carcinomas or other diseases [13]. Natural complexes have been broadly studied for the treatment of several diseases due to their diverse properties as stimulation of tumour-defeating autophagy or acting as antimicrobial agents [14].

Studies showed that caffeine and curcumin can induce autophagy in tumors [27, 54] while antibacterial effects have been demonstrated for eugenol, cinnamaldehyde, curcumin and carvacrol [55, 56]. The improvement of their bioavailability was obtained by integrating in NPs.

## Nano delivery and its application for cancer pharmacotherapeutic management

The nano delivery systems are designed to deliver diagnostic and therapeutic molecules at the targeted place. This technique has been extended studied in the last 15 years and have a major influence in personalized medicine. Proficient targeted release methods permit for a diminished systemic dose though proceeding in comparatively advanced or more competent dosing at the target location [15]. Nanoscale materials are essential for the majority of intended delivery systems as these structures can penetrate through different areas in the organism till reach the target spot. For the delivery of certain molecules at the cancer site, the particles administered IV should be small enough to can go from the blood steam through the microvasculature of the tumour that request mainly particles with diameters from 100 nm to 2 µm [56]. Nano elements are compatible materials for intended tumour delivery because of their capability to stream in the blood flow for comparatively extensive periods and their capability to build up in spaces of the tumour.

Nanoparticles could protect bioactive agents against high pH and/or metabolic degradation, thus prolonging the drug life span. Therefore, nanosized carriers could effectively modulate pharmacokinetics, enhancing drug efficacy beside reduced toxicity and offer the possibility to deliver bioactive agents in a controlled and, sometimes, site-specific manner.

Also, nanoparticles has been utilized to deliver metabolic drugs. For example, nanoparticles with the antidiabetic drug metformin have been shown to be effective in a pancreatic cancer cell population by inhibiting glutamine metabolism [13]. Other studies have shown that inhibition of metabolic pathways or RAS ptoteins, especially mutant KRAS, could be a new possibility in anticancer therapy [57].

In a research study by Wang et al. [37], the biological dispersal and performance of intended nanoparticles made up of heparin-folate-paclitaxel conjugates packed with paclitaxel (HFT-T) were contrasted to non-aimed nanoparticles of heparin-paclitaxel combinations laden with paclitaxel (HT-T). HFT-T targeted systems considerably decreased tumour volume more than Np and Paclitaxel manage in a KB-3–1 human nasopharyngeal cancer xenograft-holding mouse model. Despite this, biodistribution research studies discovered that the variation between the buildup of intended and non-intended systems in the tumour was not statistically considerable [58].

The requirement for the most developed technology to play a significant role in the management of carcinoma is noticeably apparent in the statistics representing that carcinoma frequency, prevalence, and death continued at more than high levels. Nanoparticles may be extremely useful for imaging applications [59] as of the raised surface-area-to-volume ratio in addition to comprising the prospective for several sites for chemical change that perhaps utilized to intensify the sensitivity of imaging [59]. Whereas the escaping from the uptake of macrophage is significant for nanoparticle intervened outcomes in lots of examples, the tendency of nanoparticles to go through macrophage-intervened phagocytosis may be useful for applications in imaging techniques.

Superparamagnetic iron oxide nanoparticles (SPIONs) have been utilized for magnetic resonance imaging of lymph nodes after uptake of macrophage, which will possibly help discover any spreadable cancerous disease [60, 61]. Currently, a novel approach for the bioequivalence assembly of nanoparticles with mediators of imaging was illustrated [62]. In principle, extremely particular imaging of little quantities of malevolent cells could be accomplished by connecting a targeting agent, for instance, a monoclonal antibody, with Gd^3+^-chelates to influence magnetic resonance (MR) relaxivity or combining with other imaging investigations. Sensitivity is a challenging issue of research in imaging. One prospective approach is to intensify the signal in the part of interest by conveying the appropriate enzyme. For instance, horseradish peroxidase has been transported to tumours of xenograft via conjugation to tumour-specific monoclonal antibodies, and this has been utilized to oligomerize MR-definite ligands to attain an improved signal for imaging and detection of tumour [63]. Magnetic nanoparticles can be utilized for both advanced magnetic resonance imaging and applications of hyperthermia for progressive cancer management [63]. Iron oxide nanoparticles can be combined with methotrexate [64], paclitaxel [65], or other anticarcinogenic drugs [66] for theranostic (therapeutic and diagnostic) applications. Nanoparticles of gold, quantum dots, and carbon nanotubes have also been customized and used for possible theranostic applications [67]. The manufacturing of medicine at the nanoscale level has been considered broadly. It is undoubtedly, the most progressive technology in the field of Np applications as of its possible benefits for instance the likelihood to alter properties such as bioavailability, solubility, diffusivity, medicine releasing profiles, and immunogenicity. This can therefore lead to the progress and advancement of suitable administration ways, minimum toxicity, improved bio delivery, a small number of side effects, and expanded life cycle of drug [51]. Target transport is another significant part that uses nanomaterials as drug delivery systems and is classified into active and passive transport. In active positioning, moieties, for example peptides and antibodies are combined with system of drug delivery to connect them to the structures of receptor articulated at the target location. In passive targeting, the prepared carrier compound of drug moves in the course of the blood flow. It is driven to the site of object by attraction or binding affected by properties like temperature, pH, shape and molecular site. The chief targets in the human body are the receptors present on the cell membranes, antigens or proteins and lipid contents of the cell membrane and the surfaces [68].

The combination of diagnosis and treatment is described as theranostic and is being widely used for cancer management [69, 70]. Theranostic nanoparticles can help in the diagnosis of the disease, recognizing the phase of the disease, reporting the location, and give information regarding the response of treatment. Additionally, such nanoparticles can transmit a curative agent to the tumour, which can offer the essential concentrations of the healing agent utilizing molecular and/or outer stimuli. Studies showed that the combination of alginate with folic acid-altered chitosan nanoparticles were efficient for revealing colorectal carcinoma cells using an illumination arbitrated mechanism based on the properties of nanoparticles to increase 5-aminolevulinic acid (5-ALA) liberation in the lysosome of the cell [69–71].

Hyaluronic acid is one more biopolymeric material. This is a bio-friendly, negatively stimulated glycosaminoglycan, and is one of the most important components of the extracellular matrix [72, 73]. Hyaluronic acid can combine with the CD44 glycoprotein receptor, which is frequently over-expressive in a variety of carcinogenic cells, using the receptor connecting interrelation. Therefore, hyaluronic acid-altered nanoparticles are fascinating for their utilization in the diagnosis and treatment of carcinoma [74–76]. The perspective of this procedure was examined in both the live cells and in the laboratory. Amplified uptake of nanoparticles by cancer cells was detected by magnetic resonance imaging when an outer magnetic field was utilized [77]. After the intravenous administration of the nano-medium in three milligrams per kilogram (concerning the free medicine) rats, a huge ablation of the tumour was detected. After management, the tumours nearly vanished [77, 78] made a nanoparticulate multipurpose complex system by encapsulating Fe_3_O_4_ Np in dextran Np combined to redox-responsive chlorine 6 (C6) for near-infrared and MR imaging. Hong et al. produced glioma cells or theranostic nanoparticles of C6 mice. These particles consisted of gadolinium oxide nanoparticles covered with folic acid-combined dextran or paclitaxel. The bioprotective properties of dextran covering and the chemotherapeutic outcomes of paclitaxel on the C6 glioma cells were assessed by the MTT (colorimetric) assay. The manufactured nanoparticles have been revealed to come in contact with C6 tumor cells by receptor-arbitrated endocytosis and offer improved contrast (in MR) concentration-reliant activity because of the paramagnetic property of the gadolinium nanoparticle.

## Chitosan-based nanoformulations

Many nano-polymeric systems have been constructed and characterized based on both synthetic polymers and natural polymers having their drawbacks and advantages. Natural polymers such as alginate, chitosan, and hyaluronic acid have been studied for the fabrication of nanoparticle systems (Table 2). Despite progress in the drug delivery system, oral administration of the drug is still desired.

**Table 2:** The most relevant pharmacological studies of chitosan nanophormulations

Chitosan Nanophormulations	Purpose	Findings	Refs.
Oral drugs semi-synthetic biopolymer chitosan complexes	Drug delivery developing	↑Solubility, ↑formation and ↑stability of SSBC	[79]
Rasagiline encapsulated chitosan-coated PLGA nanoparticles	Evaluation of encapsulation efficiency	↑Bioavailability in the brain	[80]
Chitosan, hydroxypropylmethylcellulose, pluronic F127, polyaniline, BCNU-Nano-co-Plex	Nose to brain drug delivery system developing	↑ Release of bioactive agent	[81]
Mnps coated with non-cross-linked chitosan	In vitro/Evaluating of rat aortic endothelial cells (ecs) viability In vivo/ evaluating issue distribution	no effect on cell viability ↑biocompatibility of MNPs	[82]
Chitosan nanospheres with methotrexate	In vitro/ evaluating of nanoparticles containing methotrexate	Sustained release, ↑ Passive targeted delivery system for MTX, ↓ Side effects of the drug	[83]
Chitosan/gelatin nanocarriers	In vitro evaluation /for calcium hydroxide delivery	↑ Release of calcium ions	[84]
Chitosan grafted halloysite nanotubes	In vitro/ evaluation of anticancer effect of curcumin on hepg2, mcf-7, sv-huc-1, ej, caski, hela cells	↑ Anticancer effect ↑ Apoptosis	[85]
Curcumin-loaded O-CMCS/n-zno nanocomposite	In vitro efficacy/ evaluation of delivery of curcumin on MA104 cells	↑ Curcumin release	[87]
Chitosan nanoparticles Chitosan beads	Betamethasone and teracycline encapsulation efficiency	Drug released from chitosan nanoparticles is lower than that released from chitosan beads	[88]
Encapsulating chitosan (CS) nanoparticles (nps)	Chemotherapeutics targeted delivery	↑ Tissue targeting ↑ Controlled drug release	[89]
Pyrazolopyrimidine, pyrazolopyridine thioglycosides encapsulated by chitosan nanoparticles	In vitro efficacy/huh-7, mcf-7 cells	↑ Anti-cancerous activity	[90]
Raloxifene-encapsulated hyaluronic acid-decorated chitosan	In vitro efficacy/ lung a549 cancer cell line	↑Cytotoxicity ↑ entrapment efficiency	[91]
Self-aggregates from deoxycholic acid-modified chitosan	Delivery vehicle of genes	↑Anti-cancer effect	[92]
Cytarabine-loaded chitosan nanoparticles	Drug delivery system	↑Anti-cancer effect	[93]
10-hydroxycamptothecine nanoneedles Methotrexate-chitosan conjugate	Dual-drug delivery system	↑Anti-cancer effect	[94]
Fe_3_O_4_/carboxymethyl-chitosan nanoparticles	Model anti-tumour drug	↑Cellular uptake ↓rapa drug damage	[95]
Encapsulated Fe3O4-blf	In vivo /mice	Complete regression of the tumour	[96]
Doxorubicin-loaded zein nanoparticles	In vitro/ cancer cells	↑anti-cancer effect	[97]
DTX-HGC nanoparticles	In vitro /A549 lung cancer cells	Antitumor efficacy	[98]
Tamoxifen nanoformulations	In vitro efficacy/ rat intestinal tissue	↑Drug permeated	[99]
Letrozole with chitosan nanoparticles	Pharmaceutical carrier	↑Anticancer efficacy	[100]
Ciprofloxacin hydrochloride-loaded nanoparticles	Drug carrier	↑Ciprofloxacin release	[101]
5-fluorouracil CS-SPION	Drug carrier	↑Drug loading efficiency	[106]
Chitosan nanospheres with 5-FU	In vitro/ HT29 and PC-3 cells	↓Tumour cell proliferation ↓HT29, PC-3 adhesion	[107]
Chitosan-coated curcumin nanocrystals	In vitro and in vivo/murine model of lps induced endotoxemia	↑Nrf2, ↑GST, ↑SOD, ↑NF-kB	[109]
Simvastatin loaded nanoparticle	In vivo/mice	↓Lipid profile	[125]

Sithole et al. [79] reviewed the novel class of biopolymers called semi-synthetic biopolymer complexes (SSBC) as nanocarriers for oral drug administration, due to their anomalous properties. The review also elucidates the complexation of some natural polymers with selected synthetic chemicals to indicate few factors that have an impact on the preparation solubility, formation and stability of SSBC. It also discusses specific significant structural and functional attributes or effects which are essential to be taken into consideration when an oral drug delivery system is developed [79].

Ahmad reported the rasagiline-encapsulated chitosan-coated poly (lactic-co-glycolic acid) (PLGA) nanoparticles (RSG-CS-PLGA-NPS) in a double emulsification-solvent evaporation technique. The mean particle size, polydispersity index and encapsulation efficiency were 122.38, 0.212, and 75.83, respectively. Consequently, intranasal delivery of the drug showed significant enhancement of bioavailability in the brain [80].

Akilo et al. prepared the BCNU-Nano-co-Plex (the bioactive agent) loaded with chitosan, hydroxypropylmethylcellulose, pluronic F127 and polyaniline. The release of bioactive agent demonstrated a 10.28% release of nanoparticles per application cycle, which may be useful as a nose to brain drug delivery system that can be modulated to deliver bioactive agents to the brain via electro-actuation [81].

Agotegaray et al. reported the MNPs (magnetic nanoparticles) consisting of magnetite functionalized with oleic acid and coated with the biopolymer chitosan and glutaraldehyde-cross-linked chitosan. After 36 h, they observed the decrease in cell viability and concluded that improved biocompatibility of MNPs, resulting in better nano-systems for targeted drug delivery [82].

Dhanaraj et al. evaluated the chitosan nanoparticles containing methotrexate for the drug delivery system. The nanoparticle was prepared by emulsion polymerization method using glutaraldehyde as the cross-linking agent. One of the samples showed the least particle size, optimum zeta potential range, moderate drug loading efficiency followed by sustained drug release over 48 h [83].

Farhadian et al. optimized the chitosan/gelatin nanocarriers (NCs) for calcium hydroxide (CH) delivery. Drug loading (DL), encapsulation efficiency (EE) and particle size were 88.5%, 99% and 292 nm respectively. FTIR analysis showed the presence of hydrogen bonding and a few other intermolecular interactions. These interactions enable chitosan/gelatin NCs to load CH and maintain a sustained release of calcium ions from CH during the experimental period [84].

Liu et al. prepared the chitosan grafted halloysite nanotubes (HNTs-g-CS). The study suggests that HNTs-g-CS are potential nanocarriers for drug delivery in cancer therapy, as curcumin loaded HNTs-g-CS increased apoptosis on EJ cells [85].

Shanmukhapuvvada and Vankayalapati developed hydrophilic polymers chitosan nanoparticles using the emulsification cross-linking method. One of their formulations exhibited release kinetics of 86.5% and entrapment efficiency of 55% indicating the prolonged period of drug-releasing capacity [86].

Upadhyaya et al. synthesised O-carboxymethyl chitosan (O-CMCS) based nanocomposites (NCs) with nanostructured zinc oxide (n-ZnO). The drug release was and controlled in the initial phase and sustained in the later phase [87].

Taghizadeh et al. synthesised chitosan nanoparticles and chitosan beads as carriers for betamethasone and tetracycline using sodium citrate as the cross-linking agent. They reported that the drug released from chitosan nanoparticles is lower than that released from chitosan beads [88].

There are many studies on the elaboration of chitosan-based nanoformulations which could be used in cancer treatment.

Abbas et al. developed an inhalable formulation consisting of chitosan (CS) nanoparticles (NPs) and CS magnetic nanoparticles (MNPs) encapsulating polyvinylpyrrolidone (PVP)/maltodextrin (MD)-based microparticles (MPs). Both CS NPs and CS MNPs were of the same size at ~ 6 μm, but the drug release was improved by a factor of 1.7 in the case of CS MNPs. This formulation showed great therapeutic improvements for drug delivery to tumours which are present in deep ling tissues [89].

The anti-metabolic compounds pyrazolopyrimidine and pyrazolopyridine thioglycosides were synthesized and encapsulated by chitosan nanoparticles to increase the anti-cancerous activity. This nanoformulation was evaluated for its cytotoxicity against Huh-7 and Mcf-7 cells which are related to liver and breast cancer cells respectively. Genotoxic effects and a synergistic effect was conducted by cellular DNA fragmentation assay and simulated on CompuSyn software [90].

Almutairi et al. prepared the raloxifene-encapsulated hyaluronic acid-decorated chitosan nanoparticles by complexation. They showed that this formulation has the highest entrapment efficiency (EE%) (92%) and induced the highest cytotoxicity against the human lung A549 cancer cell line [91].

Bae et al. prepared the self-aggregates from deoxycholic acid-modified chitosan. These self-aggregates can form complexes with these self-aggregates. They may find potential applications as a delivery vehicle of genes and anti-cancer drugs placid DNA [92].

Deepa et al. evaluated the in-vitro efficacy of. The study suggests that chitosan nano-formulation would be an efficient approach for the release of cytarabine against solid tumours and might be a better [93].

Wu et al. synthesised 10-hydroxycamptothecine nanoneedles integrated with an exterior thin layer of the methotrexate-chitosan conjugate, which is a dual drug, using co-precipitation in the aqueous phase. They concluded that the emergence of a dual-drug delivery system which enhances the therapeutic performances in cancer treatment [94].

Li et al. Synthesised the Fe_3_O_4_/carboxymethyl-chitosan nanoparticles as carrier and rapamycin as the model anti-tumour drug (Fe3O4/CMCS-Rapa NPs). Fe3O4/CMCS-Rapa NPs could enhance cellular uptake and reduce Rapa drug damage to the normal cells to improve the curative effect of the drug on tumour cells [95].

Roy et al. encapsulated Fe3O4-bLf (Fe_3_O_4_-saturated lactoferrin) in alginate enclosed chitosan-coated calcium phosphate (AEC-CP) nanocarriers (NCs). The complete regression of the tumour in triple-positive (EpCAM, CD133, CD44) was observed in 70% of mice fed on non-targeted (NT) NCs. In comparison, 30% of mice show tumour recurrence after 30 days, and only 10% of mice fed with targeted NCs showed tumour recurrence [96].

Arunkumar et al. synthesised the composite injectable chitosan gel (DZ-CGs) comprising of doxorubicin-loaded zein nanoparticles (DOX-SC ZNPs). In vitro drug release profiles of composite DZ-CGs were found to be more controlled when compared to DOX-SC ZNPs. Also, Composite DZ-CGs were more effective in killing cancer cells when compared to DOX-SC ZNPs [97].

Hwang et al. synthesised the hydrophobically modified glycol chitosan (HGC) nanoparticles loaded with the anticancer drug docetaxel (DTX). The DTX-HGC nanoparticles showed higher antitumor efficacy such as reduced tumour volume and increased the survival rate in A549 lung cancer cells [98].

Barbieri et al. prepared the nanoformulation based on phospholipid and chitosan, which efficiently loads tamoxifen by encapsulation method. The amount of drug permeated using the nano-formulation was increased from 1.5 to 90 times. This nano-formulation enhanced the non-metabolized drug passing through the rat intestinal tissue via paracellular transport [99].

Gomathi et al. fabricated the anticancer drug—letrozole® with chitosan nanoparticles using sodium tripolyphosphate as the crosslinking agent. The nano-formulation has biocompatible and hemocompatible properties which makes it an efficient pharmaceutical carrier for the anticancer drug letrozole [100].

Jain and Banerjee compared the five different drug-carrier ratios of ciprofloxacin hydrochloride-loaded nanoparticles of albumin, gelatin, chitosan, and lipid [solid lipid nanoparticles (SLNs)]. A drug-to-carrier ratio of 0.5:1 was preferred for chitosan nanoparticles having a zeta potential of > 20 mV and drug encapsulation of 35%. Their results suggest that chitosan nanoparticles and SLNs can act as promising carriers for sustained ciprofloxacin release in infective conditions [101].

Khan et al. prepared temozolomide® loaded nano lipid-based chitosan hydrogel (TMZNLCHG) by encapsulation method. The study revealed the formulation of a non-invasive intranasal route for brain targeting as an alternative to another route for TMZ [102].

Wang and Zhao optimized the preparation of anticancer drug—gefitinib® and chitosan protamine nanoparticles. The best formulation of gefitinib—chitosan protamine nanoparticles was 1 mg·mL^−1^ of gefitinib, 3.5 mg·mL^−1^ of chitosan and 1 mg·mL^−1^ of protamine with a drug loading of 19.55% [103].

Koo et al. prepared the water-insoluble paclitaxel encapsulated into glycol chitosan nanoparticles with hydrotropic oligomers (HO-CNPs). Paclitaxel-HO-CNPs showed higher therapeutic efficacy, compared to Abraxane®, a commercialized paclitaxel-formulation [104].

Maya et al. prepared the O-carboxymethyl chitosan (O-CMC) nanoparticles, surface-conjugated with cetuximab (Cet) for targeted delivery of paclitaxel. They observed the Cet-Paklitaxel-O-CMC nanoparticles are a promising candidate for the targeted therapy of epidermal growth factor receptor (EGFR) overexpressing cancers [105].

Al-Musawi et al. synthesised prepared chitosan-covered superparamagnetic iron oxide nanoparticles (CS-SPION) and applied them as a nano-carrier for loading of (5-FU) (CS-5-FU-SPION). The remarkable drug loading efficiency (~ 73%) was notable. FA-CS-5-FU-SPION demonstrated sustained release of 5-FU at 37 °C in both phosphate and citrate buffer solutions using a reverse microemulsion technique. There were no adverse outcomes reported for normal cells and observed that fluorescein isothiocyanate-labelled drug, has an effective entrance into a cancerous cell and stimulate cell death and apoptosis [106].

Cavalli et al. prepared chitosan nanospheres with 5-FU by a combination of coacervation and emulsion droplet coalescence method. The encapsulation efficiency was ~ 70%, and the %age of 5-FU delivered from nanospheres was ~ 10% after 3 h. Thus, nanospheres were effective in reducing tumour cell proliferation and were able to inhibit both HT29 and PC-3 adhesion to HUVEC after 48 h of treatment [107].

Sahu et al. prepared 5-FU loaded biocompatible chitosan nanogels (FCNGL) using the ion gelation technique. The pH-responsive character of nanogels triggered the release of 5-FU in an acidic environment, resulting in selective drug delivery, leading to sustained delivery of 5-FU for chemotherapy that can result in high efficacy, patient compliance and safety [108].

The potential of intracorporeal chitosan-coated curcumin nanocrystals (Chi-CUR-NC-4b) were examined as a therapeutic application against endotoxemia-induced sepsis. The fabricated nanocrystals were assessed for pharmacokinetic and pharmacodynamic parameters. Chi-CUR-NC 4b was ascertained to neutralise lipopolysaccharide (LPS) and increased plasma drug concentration with enhanced levels in the lungs and liver. In vitro and in vivo pharmacodynamic studies implied that the defensive effects were mediated by the up-regulation of Nrf2 (enhanced antioxidant activity, i.e. via elevated levels of Glutathione-S-transferase (GST) and Superoxide Dismutase (SOD) as well as the downregulation of nuclear factor kappa-light-chain-enhancer of activated B cells (NF-kB). NRF2 has been implicated in creating chemoresistance and has been linked to RAS driven cancer [16, 17]. These effects lead to decreased cytokine secretion and decreased tissue injury resulting in enhanced survival in the murine model of LPS induced endotoxemia [109].

Anitha et al. prepared the nanoformulation of curcumin using dextran sulphate and chitosan. The results showed the preferential killing of cancer cells compared to normal cells by the curcumin-loaded drug [110].

Baghbani et al. prepared the curcumin-loaded chitosan/perfiuorohexane nanodroplets using a nanoemulsion process. The curcumin entrapment was 77.8%. The sonication at a frequency of 1 MHz, 2 W/cm^2^ for 4 min triggered the release of 63.5% of curcumin from optimal formulation [111].

Keerthikumarc et al. synthesised chitosan encapsulated curcumin nanoparticles by ionic gelation method. Chitosan nanoparticles formulations showed sustained release of the drug; also, in vitro cytotoxicity study showed high and long term anticancer efficacy in human oral cancer cell lines till 72 h [112].

Rajan et al. synthesised curcumin nanoparticles loaded in chitosan biopolymer and bovine serum albumin. They observed that the selective drug targeting of colorectal carcinoma cells was effective when concentration was increased [113].

Moreover, there are also studies on the testing of chitosan nanoparticles with plant extracts. Shahiwala et al. synthesised the chitosan nanoparticles with alcoholic extract of *Indigofera intricate—*plant of potential antitumor properties*.* Almost a 500-fold reduction in the extract concentration required to achieve the same anticancer activity when formulated as nanoparticles [114].

Alipour et al. studied the sustained release of silibinin-loaded chitosan nanoparticles (SCNP). They reported the positive zeta potential of nanoparticles were + 11.5, and cytotoxicity assay indicated that drug formulation was toxic to C6 glioma cells [115].

George et al. studied the functionalised nanohybrid hydrogel using L-histidine (HIS) conjugated chitosan, phyto-synthesised zinc oxide nanoparticles (ZNPs) and dialdehyde cellulose (DAC) as a sustained drug delivery carrier for the polyphenol, plant-derived compounds—naringenin, quercetin and curcumin. Anticancer studies towards A431 cells (epidermoid carcinoma) exhibited excellent cytotoxicity with a 15 to 30-fold increase using the hybrid carrier, compared to the free polyphenol drugs [116].

The chitosan nanoparticles are also tested for other groups of drugs. For example anti-inflammatory drugs. Agotegaray et al. reported that the nanodevice consists of a magnetite core coated with chitosan (Chit@MNPs) as a platform for diclofenac loading as a model drug and observed the marginal variation in the efficacy [117]. Chaichanasak et al. prepared the chitosan-based nanoparticles with damnacanthal (DAM). DAM increased the levels of the tumour suppressor non-steroidal anti-inflammatory drugs-activated gene 1 in the nucleus, therefore causing improved anticancer effects [118].

There are also studies on antifungal and antibacterial drugs. Calvo et al. prepared the chitosan nanocapsule comprising tioconazole (TIO) and econazole (ECO) by encapsulation method. The association efficiency was 99% for TIO and 87% for ECO. The drug showed fungicidal activity against *C. Albicans* at non-toxic concentrations and reported it as the first step in the development of a pharmaceutical dosage for treating vaginal candidiasis [119].

Abd Elsalam et al. proposed a novel chitosan-based nano-in-microparticles (NIM), which acts as a combination therapy in the antibacterial platform. PEGlyation (PEG—polyethene glycol) was done on chitosan, which increased its solubility in water. To treat multiple bacterial strains, the antibacterial activity of the PEG-CS was strengthened using immobilized silver nanoparticles and with dendritic polyamidoamine hyperbranches. Ibuprofen encapsulated by montmorillonite nanoclay (MMT) was used as an anti-inflammatory drug. The developed drug showed good antibacterial activity against both aerobic and anaerobic bacteria resulting in treating multiple bacterial infections [120].

Ciprofloxacin, a broad-spectrum antibiotic; a poorly soluble drug-loaded chitosan nanoparticle, was prepared for the therapeutics of various microbial infections. Nanoformulation of ciprofloxacin was developed using 85% deacetylated chitosan as a biodegradable polymer and tripolyphosphate (TPP) as a cross-linking agent by ionotropic gelation technique. The Fourier Transform Infrared Spectroscopy (FTIR) studies showed that there was zero interaction found between the drug ciprofloxacin and chitosan. One of the formulations was found to have good entrapment efficacy, positive zeta potential value, and its size was from 100 to 200 nm [121].

Manimekalai et al. prepared the ceftriaxone sodium loaded chitosan nanoparticles using chitosan as a polymer and trisodium polyphosphate as a cross-linking agent. The chitosan nanoparticles developed was capable of sustained delivery of ceftriaxone sodium [122].

Jamil et al. prepared the cefazolin loaded chitosan nanoparticles (CSNPs) by ionic gelation method. Kinetics study had demonstrated the excellent antimicrobial potential of cefazolin loaded CSNPs against multidrug-resistant *Klebsiella pneumoniae, Pseudomonas aeruginosa* [123].

Moreover, Manuja et al. synthesised the chitosan/mannitol quinapyramine sulfate (QS) nanoparticles (ChQS-NPs) which could be used as a trypanocidal agent in veterinary. They concluded the ChQS-NPs are safe, less toxic and effective as compared to the conventional QS drug delivery [123].

Among other drugs combined with chitosan nanoparticles, it is noteworthy to mention that are also studied antihypertensive, antidepressant and eye droop formulations.

Niaz et al. fabricated the antihypertensive (AHT) nano-carrier systems (NCS) encapsulating captopril, amlodipine and valsartan using chitosan (CS) polymer. They reported that the AHT nano-ceuticals of polymeric origin can improve the oral administration of currently available hydrophobic drugs while providing the extended-release function [124].

Selvasudha and Koumaravelou prepared chitosan on simvastatin loaded nanoparticle. Better absorption was observed by reducing the lipid profile with several-fold reduced dose in the mouse model. Studies revealed possible synergistic functionalities of chitosan and the simvastatin as potential hypolipidaemic modality without any toxic manifestations [125].

Dhayabaran et al. encapsulated antidepressant drugs with biopolymer chitosan. Synthetic drug (venlafaxine) and herbal extracts (*Hypericum perforamtum* and *Clitoria ternatea*) were encapsulated. They developed a strategy against depression by utilizing the potentials of *Clitoria ternatea* as a drug in nanomedicine [126].

Yu et al. prepared water-soluble cerium oxide loaded glycol chitosan nanoparticle for the treatment of dry eye disease. The solubility of cerium in GC (GCCNP) increased to 709.854 μg/ml compared to its original solubility (0.020 μg/ml) in cerium oxide. Concluded that GCCNP can be the potential drug in the form of eye drop for the treatment of dry eye [127].

## Application of chitosan in nanomedicine—pre-clinical and clinical studies

The performed scientific studies have provided promising results of chitosan nanoparticles in the anticancer drug delivery and oncological treatment (Tables 3, 4).

**Table 3:** Application of chitosan nanoparticles in the nano-drug delivery system in pre-clinical and clinical studies

Compound used in combination with chitosan nanoparticles	Features	References
Pre-clinical studies		
Bupivacaine	↑ Anaesthetic effects of bupivacaine	[150]
Paclitaxel	↑Tumour-targeting effect	[136]
Prothionamide	↑ Treatment of tuberculosis	[148]
Hydrocortisone	↑Elastic connectivity of tissues which improves atopic dermatitis	[149]
Curcumin	↑ Curcumin’s anticancer activity against colon and breast cancer cells	[145]
Albumin	↑ Oral delivery of albumin	[146]
*Ocimum gratissimum* essential oil	↑Antibacterial activity ↑anticancer activity against breast cancer	[144]
Triphosphate	Improve delivery and health effects	[143]
RGD peptides	Localize to the tumour vasculature ↑antiangiogenic effects	[142]
Clinical studies		
Morphine	Improve pain medications of morphine	[151]
Doxorubicin	↓ Doxorubicin toxicity ↓ tumour growth	[141]

**Table 4:** Toxicity of chitosan and chitosan derivatives in descending order of degree of deacetylation (DD)

Chitosan proprieties (DD, MW)	Modification	Methods	IC_50_ value	Refs.
100% DD, 100 kDa 36%	Trimethyl chitosan, chloride salt	In vitro, MCF7 cells	0.83 ± 0.325 mg/mL	[167]
100% DD, 100 kDa 36%	Trimethyl chitosan, chloride salt	In vitro, COS7 cells	> 10 mg/mL	
100% DD, 152 kDa	Glycol chitosan	In vitro*,* B16F10 cells	2.47 ± 0.15 mg/mL	
100% DD, 3–6 kDa	20, 44, 55% Trimethyl chitosan, chloride salt	In vitro, MCF7, COS7 cells	> 10 mg/mL	
100% DD, 3–6 kDa	94% Trimethyl chitosan, chloride salt	In vitro, MCF7 cells	1.402 ± 0.210 mg/mL	
100% DD, 3–6 kDa	94% Trimethyl chitosan, chloride salt	In vitro, COS7 cells	2.207 ± 0.381 mg/mL	
97% DD, 65 kDa	N-octyl-O-sulphate	In vivo, i.v., mice	102.59 mg/kg	[168]
97% DD, 65 kDa	N-octyl-O-sulphate	In vivo, i.p., mice	130.53 mg/kg	
97% DD, 65 kDa	N-octyl-O-sulphate	In vitro, primary rat hepatocytes	> 200 mg/mL	
95% DD, 18.7 kDa	Steric acid conjugation micelle	In vitro, A549 cells	369 ± 27 μg/mL	[169]
95% DD, 18.7 kDa	Steric acid conjugation and entrapment in micelle		234 ± 9 μg/mL	
87% DD, 20, 45, 200, 460 kDa	None, Lactic acid salt	In vitro, Caco-2 cells	0.38 ± 0.13, 0.31 ± 0.06, 0.34 ± 0.04, 0.37 ± 0.08 mg/mL	[170]
87% DD, 20, 45, 200, 460 kDa	None, hydrochloride salt		0.23 ± 0.13, 0.22 ± 0.06, 0.27 ± 0.08, 0.23 ± 0.08 mg/mL	
87% DD, 20, 45, 200, 460 kDa	None, glutamic acid salt		0.56 ± 0.10, 0.48 ± 0.07, 0.35 ± 0.06, 0.46 ± 0.06 mg/mL	
87% DD, 20, 45, 200, 460 kDa	None, aspartic acid salt		0.67 ± 0.24, 0.61 ± 0.10, 0.65 ± 0.20, 0.72 ± 0.16 mg/mL	
85% DD, 60–90 kDa	None, hydrochloric acid salt	In vitro*,* B16F10 cells	2.24 ± 0.16 mg/mL	[171]
84.7% DD, 400, 100, 50, 25, 5 kDa	40% Trimethyl chitosan	In vitro, L929 cells	> 1000 μg/mL	[172]
84.7% DD, 300 kDa	6.44% PEG-modified 40% trimethyl chitosan (and all PEG-modified TMC with lower Mw)		> 500 μg/mL	
84.7% DD, 3.6 MDa	25.7%PEG modified 40% trimethyl chitosan		370 μg/mL	
84.7% DD, 1.89 MDa	12% PEG modified 40% trimethyl chitosan		220 μg/mL	
81% DD, 100–130 kDa	None, hydrochloric acid salt	In vitro, B16F10 cells	0.21 ± 0.04 mg/mL	[171]
78% DD, 80% DD, 60–90 kDa	None, glutamic acid salt		2.47 ± 0.14 mg/mL	
77% DD, 180–230 kDa	None, lactic acid salt		1.73 ± 1.39 mg/mL	

*DD* degree of deacetylation, *MW* molecular weight

Nevertheless, nowadays chitosan nanoparticles clinical applications for diagnosis and therapy of cancer has been discussed because of their minimal systemic toxicity both in vitro and some in vivo models and maximal cytotoxicity against cancer cells and tumours [33, 128]. Nano drug delivery systems based on chitosan nanoparticles have been developed for pre-clinical and clinical studies [129]. Translation of novel nano-drug delivery systems from the bench to the bedside may require a collective approach.

Chitosan nanoparticles typically characterized by a positive surface charge and mucoadhesive capacities such that can adhere to mucus membranes and release the drug payload in a sustained release manner [33]. Due to such characteristics of chitosan nanoparticles their applications consist of per-oral delivery, ocular drug delivery, nasal drug delivery, pulmonary drug delivery, mucosal drug delivery, gene delivery, buccal drug delivery, vaccine delivery, vaginal drug delivery and cancer therapy [130]. The clinical studies have shown that intravenous administration of chitosan-based nanocarriers for brain delivery and intranasal administration has been an alternative due to its mucoadhesive properties, improving the patient adhesion to therapy [131] (Table 2).

Various materials with different structural forms are conjugated with drugs to prepare nano-drug delivery systems. Considering recent approaches, the most commonly used drug delivery vehicles include liposomes [132], nanoparticles (ceramic, metallic and polymeric) [133], dendrimers [134] and micelles [135]. The self-assembled amphiphilic micelles based on chitosan and polycaprolactone were developed as carriers of paclitaxel to support its intestinal pharmacokinetic profile [136]. Experimental results showed that chemical modification of chitosan nanoparticles can improve their use for therapy application [137] and improve tumour targeting [138] (Table 2).

Chitosan nanoparticles have shown anticancer activity in vitro and in vivo. Xu et al., 2009 [139] has been suggested that chitosan nanoparticles dose-dependent tumour suppression was correlated with the inhibition of tumour angiogenesis. Also, Chitosan nanoparticles can be used to deliver siRNA targeting key components of tumor metabolism Due to their low or non-toxicity, chitosan nanoparticles and their derivatives can serve as a novel class of anti-cancer drug [139] (Table 2).

Chitosan nanoparticles can be used as carriers in the controlled drug delivery of doxorubicin, an anticancer drug used for the treatment of several tumours [18]. Doxorubicin can be toxic at some points and to protect patients from doxorubicin side effects were developed chitosan nanoparticles drug delivery system. It is possible to encapsulate and deliver doxorubicin with reduced side effects. The chitosan oligosaccharide conjugated with biodegradable doxorubicin with farther high efficiency in the tumour growth suppression because of higher cellular uptake [140, 141] (Table 2).

Chitosan nanoparticles decorated with RGD peptides localize to the tumour vasculature and exert antiangiogenic effects [142]. Another composition of chitosan nanoparticle was prepared by ionic crosslinking of N-trimethyl chitosan (TMC) with tripolyphosphate with a lower degree of quaternization and an increase in particle size, a decrease in zeta potential and a slower drug-release profile. For example, ATP, a related derivative of triphosphate, is essential for life and use its encapsulation with chitosan nanoparticles can improve delivery and health effects. Such specific characteristics of N-trimethyl chitosan chloride nanoparticles can support the use of them as potential protein carriers in various modifications [143]. Pre-clinical studies with chitosan and N,N, N-trimethyl chitosan nanoparticle encapsulation of *Ocimum gratissimum* essential oil exhibited antibacterial activity at a lower concentration for both Gram-negative and Gram-positive food pathogens. In vitro cytotoxicity revealed the increased toxicity of N, N, N-trimethyl chitosan nanoparticle encapsulated in *Ocimum gratissimum* essential oil on MDA-MB-231 breast cancer cell lines [144]. Another collection approach of a nano drug delivery system based on a combination of chitosan nanoparticles with curcumin loaded dextran sulfate was studied regarding the promotion of curcumin anticancer activity. In vitro cytotoxicity measurements demonstrated that curcumin loaded polymeric nanoparticles got significant therapeutic efficacy against colon (HCT-116) and breast (MCF-7) cancer cells compared with free curcumin [145] (Table 2).

It was studied the use of chitosan nanoparticle for albumin delivery for its use as a plasma expander in critically ill patients and several other clinical applications mainly via intravenous infusion. Sustainable albumin release over time and high enzymatic stability from albumin-loaded nanoparticles were observed compared to the free albumin [146]. The chitosan nanoparticles in the nano-system delivery in combination with hyaluronic acid can be a very promising injectable system for the controlled release of platelet-derived growth factor for tissue engineering applications, as well as for the treatment of ischemia-related diseases [147] (Table 2).

Pre-clinical studies based on development and in vitro and in vivo evaluation of chitosan nanoparticles based dry powder inhalation formulations of prothionamide revealed a dose in pulmonary administration, which will improve the management of tuberculosis [148]. Hussain et al. had explored the histological and immunomodulatory actions of chitosan nanoparticle in the transport of hydrocortisone using chitosan nanoparticles against atopic dermatitis. It was shown the significant capability of chitosan nanoparticles to minimize the severity of atopic dermatitis. Histological analysis revealed that chitosan nanoparticles inhibited the elastic fibres fragmentation and fibroblast infiltration. Further, depicting their clinical importance in controlling the integrity of elastic connective tissues which makes such nanoparticles-based drug transport effective [149] (Table 2).

Bupivacaine is a long-acting local anaesthetic that belongs to the amino-amide class which is widely used during surgical procedures and for postoperative pain. Animals and in vivo studies such as infraorbital nerve blockade, local toxicity, and pharmacokinetics were used to discover the use of combination chitosan nanoparticles with bupivacaine. Pre-clinical studies bupivacaine in chitosan nanoparticles revealed that encapsulation of bupivacaine prolongs the local anaesthetic effect after infraorbital nerve blockade and altered the pharmacokinetics after intrathecal injection [149]. Currently in phase 3 clinical trials in the US and phase 2 clinical trials (UK and EU) is the chitosan-based nasal formulation of morphine (RylomineTM) [150] (Table 2).

## Stability and bioavailability of drug nanoencapsulation with chitosan

Due to its biocompatibility, biodegradability and low toxicity, chitosan is widely recognized as a safe material in pharmaceutical nanotechnology. Moreover, its versatile capabilities indicated this natural polymer and its nanoparticles as a viable vehicle in drug delivery. Once identified as an ideal drug carrier, chitosan has been exploited to design formulations for a large range of drug molecules including proteins, plasmid DNA, and oligonucleotides. Pharmaceutically, this vehiculation strategy has been successfully applied to deliver a different class of drugs such as anticancer agents, anti-inflammatory, growth factors, proteins/peptides, antibiotics and other drugs, but also in vaccine and gene therapy. Production and clinical development of nanoparticles for gene delivery are discussed nowadays. Gene therapy is an auspicious strategy with intentionally altering the gene expression in pathological cells for the treatment of gene-associated human diseases. Its discussed role of chitosan nanoparticles as a very promising carrier for gene delivery due to high biocompatibility and close resemblance to the lipidic membranes, which facilitate their penetration into the cells [152]. Furthermore, chitosan nanoparticles allow a controlled and, sometimes, site-specific delivery and are suitable to many routes of administration, especially for the non-invasive ones like oral, nasal, ocular and transdermal [153].

**Fig. 3:**
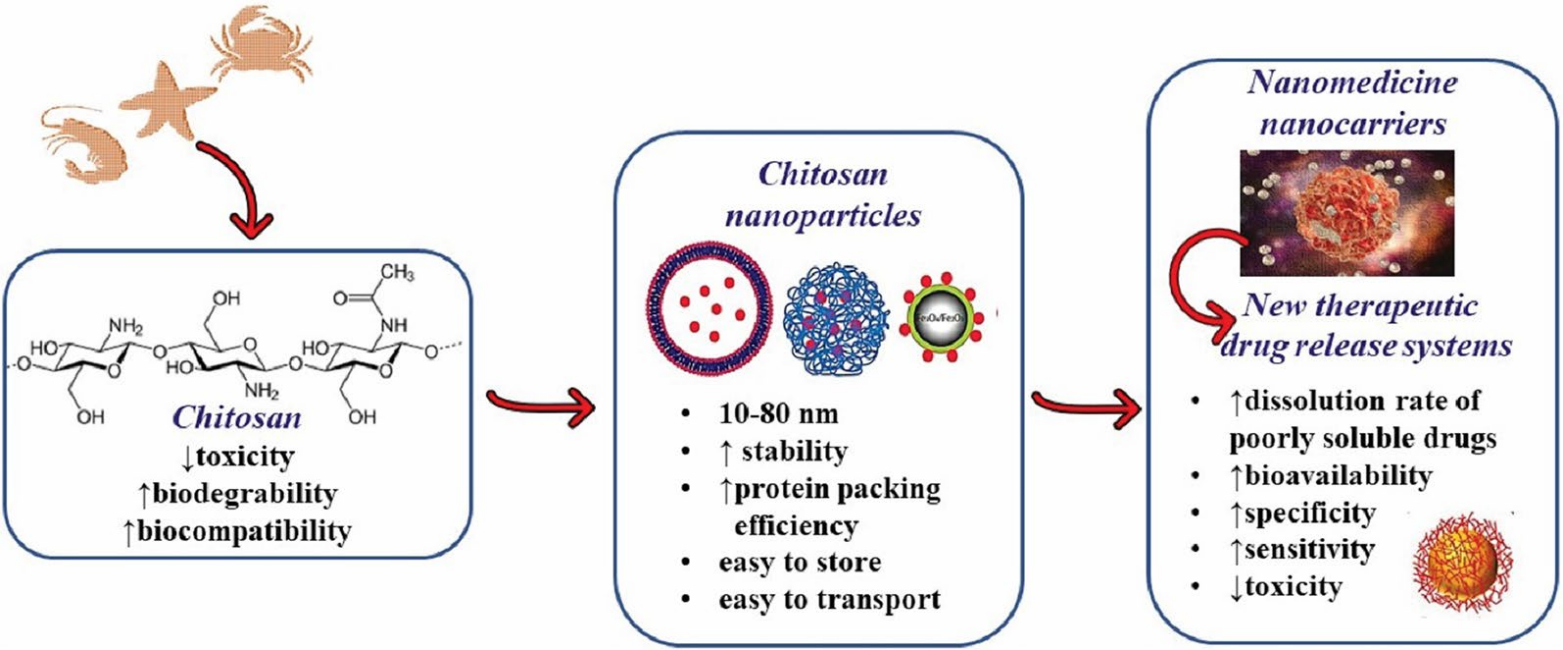
The most important advantages of chitosan nanoencapsulation

The major advantage offered by chitosan-based nanoencapsulation is the ability to improve the dissolution rate of poorly soluble drugs thus increasing their bioavailability (Fig. 3). This capability depends on the size of the particles as well as from the specific features of chitosan, which render this polymer an ideal drug carrier.

Chitosan is soluble in an aqueous solution but it possesses readily modifiable pH-responsive solubility. Generally, dissolution happens in dilute aqueous acid solutions, where the amino groups of chitosan become protonated. However, many other factors contribute to controlling solution properties such as the distribution and number of acetyl groups along the chains, pH, the ionic concentration, the conditions of isolation and drying [154].

Additionally, chitosan presents mucoadhesive and absorption-enhancing properties. The mucoadhesive nature of chitosan depends on electrostatic interaction between the positive charge on the ionizable protonated amine group and the negative charge on the mucosal surfaces. These interactions trigger a reversible structural reorganization in the protein-associated tight junctions which opens the tight junctions between cells, allowing the drug to cross the mucosal cells [155]. Mucoadhesion also extends the contact of the drug with the mucosal layer, and allow site-specific administration, in particular in those body site presenting specific mucosal surfaces such as buccal and nasal cavities. Again, many factors can influence mucoadhesive properties such as the molecular weight, the flexibility of the chitosan chain, the electrostatic interaction, the availability of hydrogen bond formation, and the capacity of spreading into the mucus due to surface energy properties [156].

Also, nanosized formulations are characterized by a large surface to volume ratios, which intensely strengthen the intrinsic properties of chitosan. Nanostructure of appropriate size and surface charge can improve drug penetration thus improving uptake through the cell membrane. Moreover, nanocarriers could protect bioactive agents against high pH and/or metabolic degradation, thus prolonging the drug life span. Therefore, nanosized carriers could effectively modulate pharmacokinetics, enhancing drug efficacy beside reduced toxicity [157] and offer the possibility to deliver bioactive agents in a controlled and, sometimes, site-specific manner.

However, there are several challenges in the use of drug nanocarriers such as low drug encapsulation, premature release, poor permeability and instability, which could finally affect drug bioavailability.

In particular, stability represents one of the most important factors regulating the efficiency of drug delivery systems, especially in the case of nanoparticles [158]. As regards chitosan nanocarriers, instability could depend on degradation by digestive enzymes and pH variation throughout the gastrointestinal tract. Additionally, a surface charge strongly influences stability and distribution and limits there in vitro and in vivo application. Indeed, although positively charged particles are strongly attracted by negatively charged cell membranes leading to an efficient internalization in the cells, the interaction with serum components could lead to severe aggregation followed by a fast clearance from the circulatory system [159]. Therefore, many attempts of tailoring the chitosan nanoparticles have been accomplished, aiming to confer improved stability against aggregation in biological settings. The most frequent strategy followed consists of hydrophilic modifications with molecules able to improve stability and solubility in slightly acid and neutral media such as β-cyclodextrin, succinic anhydride or PEG. Besides, also surface decoration with hydrophilic polymers has been carried out in the attempt to contrast nanoparticles aggregation [160].

However, changes in stability and aggregation of chitosan nanoparticles could also happen during storage. Different techniques of drying (i.e. lyophilization and spray-drying) were applied to aim to both retain the stability of the nanoparticles’ features and protect labile bioagents. In aqueous media, nanoparticles could be subjected to different processes such as solubilisation and/or degradation, drug leakage, desorption or degradation. Generally, nanopowder is easily re-dispersible, but occasionally aggregation or irreversible fusion of particles occurs making the redispersion more difficult. In this regard, the addition of bioprotectants could reduce surface attraction maintaining the nanoparticles dispersed [157].

## Physical properties and potential toxicity of chitosan-based nanomaterials drugs delivery

Chitosan is a linear polysaccharide composed of D-glucosamine units (deacetylated units) and N-acetyl-d-glucosamine units β- (1–4) -connected. Chitosan is deacetylated chitin (Fig. 1), a structural modification of chitin often carried out by alkaline hydrolysis. Commercially chitosan is produced by deacetylation of chitin, a natural material, widespread in the world of exoskeletal crustaceans. It has some remarkable therapeutic properties such as blood coagulation, fat binding, heavy metal ion complexation, hemostatic action.

The process itself used during hydrolysis causes chitin deacetylation and consequently, commercially available chitin samples have between 70 and 100% degree of deacetylation. In addition to the degree of deacetylation for a given chitosan sample, the molecular weight of the macromolecule, which can vary between 150,000–600,000 daltons, is also characteristic.

Chitin and chitosan are of high commercial interest due to their high nitrogen content (6.89%), which allows them to be used as chelating agents. Both chitin and chitosan are biodegradable, biocompatible, non-toxic, non-allergenic and renewable biomaterials and find their application in fields such as medicine, perfumes and cosmetics, food industry and agriculture [161].

Chitosan, due to the presence of the primary amine group in the sugar units form the polymeric structure, dissolves in dilute organic acids, but is insoluble in water, above pH 6–7 and in ordinary organic solvents. The solubility of chitinous substances is usually associated with the crystallinity of the sample. Higher crystallinity suggests greater or increased molecular interactions between the polymer chains. A chitinous chemical can be dissolved only if these interactions are cancelled. The intra- and intermolecular hydrogen bonds of the polymer chains are the major cause of these interactions and play an important role in the low solubility of these substances. However, chemical modifications of chitosan result in derivatives that are water-soluble in a broader pH range, including in strongly basic environments. The modifications consist of the introduction of ionic groups or substituents in the polymeric structure, which dissolves in polar solvents such as water through polar-polar interactions and determines the solubility of the macromolecule [155].

The process of isolation of chitin begins in the marine food industry. One of the by-products of this process, such as carapace of radishes, shrimps, etc., are first crushed to the powdered consistency to achieve a larger surface for the heterogeneous processes that will follow. Initial treatment of carapaces with 5% hydroxide sodium dissolves several proteins leaving chitin, lipids and calcium salt (mainly in the form of CaCO_3_). By treating with 30% hydrochloric acid, lipids are hydrolyzed, calcium salts (demineralization) and other minor inorganic constituents are broken down. Thus, the chitin obtained can be hydrolyzed using 50% sodium hydroxide at elevated temperatures to obtain chitosan. Alternatively, if isolation of chitin is not desired, the sequence based on acid treatment may be reversed to produce chitosan directly. In this method, crushed carapaces are first treated with 5% hydrochloric acid to remove calcium salts, a process often followed by the removal of proteins and lipids by treatment with 40% sodium hydroxide at higher temperatures. During the treatment with basic medium, concomitant hydrolysis of the acetamide groups of chitin takes place, the result being the formation of chitosan. The physical properties of chitinous substances are governed by two factors: the degree of deacetylation and the molecular mass.

The former has a direct impact on the secondary structure of the polymer chain and can influence and solubility of the polymer in organic or aqueous solvents. It can also affect the chemical reactivity of the sample inhomogeneous processes [162].

According to a selective nomenclature, chitinous substances that do not dissolve in dilute organic acids (e.g. 1–2% acetic acid) are called chitin, a polymer with a low degree of deacetylation. On the other hand, chitinous substances that dissolve in dilute aqueous acids are called chitosan. Solubility in aqueous acid solutions is achieved by deacetylation to the extent of 60%. However, at the degree of deacetylation between 50–60%, the distribution of the remaining acetyl groups, grafted along the polymer chain, influences the solubility of the sample. A distribution of acetyl groups on the polymer structure results in homogeneous processing conditions and gives solubility of polymers in aqueous solutions of weak acids. Instead, under heterogeneous processing conditions, polymers are formed with distinct blocks of acetylated sugar residues and are not soluble in solvents. similar. The molecular weight of chitosan obtained at the end of the production process depends on the process parameters, time, temperature and HCl and NaOH concentration. The process parameters used in chitosan production are drastic and the cleavage of the chitin structure accompanies the process. The degradation of the chitinous chain can be extended. In one preparation, a chitin sample with a molecular weight of 1.03·10^6^ kDa produced chitosan with a weight of 1·10^5^ kDa. However, the charged nature of chitosan tends to form free aggregates and the differences in the degree of deacetylation for different chitosan samples require careful implementation of the constants [163].

Many applications of any chemical, natural or synthetic, require chemical process ability. Thus, chitosan, a white powder, is difficult to handle due to the problems of solubility in neutral water, bases and organic solvents. The pKa value of the primary amino groups in chitosan is 6.5. Even if chitosan and its derivatives are soluble at a pH lower than 6, most of its applications in the basic or neutral environment cannot be achieved [164].

On the other hand, acidic solutions in which chitosan is soluble are not compatible with many applications, such as those in cosmetics, medicine and nutrition. There are two approaches in the literature on improving the solubility of chitosan at neutral pH. The first is the chemical derivatization of chitosan (for example with substituents containing quaternary ammonium group, by carboxymethylation or sulfation) so that the added substituent is hydrophilic. The second method uses chitosan with 50% diacetylation prepared by homogeneous processing of chitin. Under the conditions of homogeneous processing, the obtained chitosan remains in solution after neutralization and no derivatization is required.

Some applications of chitosan use derivatized forms thereof and to improve the solubility it is necessary to introduce ionic groups in the polymeric structure [152].

Traditionally, chitinous substances are used in rudimentary medicine and the treatment of wastewater. In recent decades, these substances have found their applicability in various fields, from textile engineering to photography. Chitosan and its derivatives have attracted more interest than chitin, even though the latter has found its applicability in medicine, fibre, absorbable tissues and bandages. It is interesting to note the resistance of chitinous substances to bile, pancreatic juice and urine, which leads to their use in surgery, but also the manufacture of human-made fibres for hard materials [95].

These substances may be subject to degradation with lysozyme, an enzyme found in nature and the human eye, and with chitinase. This has also led to the use of chitosan derivatives in the preparation of cleaning solutions for contact lenses to remove enzyme deposits [165].

Chitosan has antimicrobial properties (antibacterial and antifungal). Antibacterial action is rapid and eliminates bacteria within hours. Moreover, its derivatives are biodegradable and exhibit reduced toxicity in mammalian cells. The antibacterial activity is associated with the length of the polymer chain and suggests a cooperative effect of the individual carbohydrate units. The antibacterial property of chitosan is useful in medicine, where it is used in the manufacture of surgical accessories such as gloves, bandages, etc. It is also used to remove pathogens from water and as a food preservative by adding a layer to the outside of fruit and vegetable products [166].

As chitosan is obtained by deacetylation (usually not complete) of chitin, studies related to the analytical characterization of chitin and chitosan are not without interest. As can be seen from the structures below, the two substances differ in the presence (in the case of chitin) and the only sporadic presence (in the case of chitosan) of the acetyl group grafted by the amino function. Chitosan is immiscible with water. Some chitosan components contain hydroxyl group components, capable of intermolecular hydrogen bonds, due to the macromolecular character of the compound and due to the many intermolecular hydrogen bonds, even in the solid-state of the sample. It is difficult to discuss the toxicity of this substance, because chitosan is a natural, non-toxic and biodegradable compound, widely used, due to its unique properties, in biotechnology, human and veterinary medicine, but also cosmetics.

Chitosan is widely regarded as being a non-toxic, biologically compatible polymer. It is approved for dietary applications in Japan and many countries from Europa and the FDA has approved it for use in wound dressings. The modifications or degree of deacetylation (DD) made to chitosan could make it more or less toxic and any residual reactants should be carefully removed. A synopsis of toxicity chitosan's reported is shown in Table 4.

The toxicity of chitosan drug administration in animals was reported [173]. For the reasons listed above, the analytical use of IR spectra was passed, in the spectral range 4000–400 cm^– 1^ respectively 200–400 cm^– 1^, in the transmittance form vs. wave number. The bands are generally large due to the macromolecular character of the compound and due to the numerous intermolecular hydrogen bonds, manifested even in the solid-state of the sample. The absorption bands can be easily attributed to molecular fragments: the dominant band with a maximum at 3450 cm^– 1^ is due to the valence vibrations (stretching, *ν O–H* and *ν N–H*) of the O – H and N – H connections involved. intense in hydrogen bonds. the band with maximum absorption at 2870 cm^– 1^ is due to the valence vibrations of the C – H connections. The series of bands between 450 cm^– 1^ and 1750 cm^– 1^ are characteristic of the amide group (the bands "amide I", … "amide VI"). The production of the "amide I" band is due to a vibration mode of the amide group in which the periodic elongation of the C = O bond dominates. Because this band is associated with the acetyl groups in the molecule, its use is warranted to specify the degree of deacetylation of a chitosan sample (the more advanced the acetylation degree, the more intense this band is).

To be able to use the intensity of the "amide I" band, the spectra obtained at different recordings must be standardized. The normalization can be achieved by bringing (by mathematical processing) the intensity of the maximum band *ν O–H* and *ν N–H* to the value 1. After this operation, the integral intensity of the band “amide I” (calculated between 1750 and 1510 cm^– 1^) is dependent on the significant manner of the acetylation degree of the substance in the sample.

According to the information studied the possible cases of toxicity may arise due to the chemical transformations to which chitosan is subjected, more precisely the Degree of deacetylation (DD)**.**

## Overall conclusions

The importance of nanotechnology, in the target delivery of drugs using nanotechnologies and its application for the discovery and development of new oncological drugs are topics of great importance. The latest studies on chitosan-based nanomaterials have shown the high utility of this polymer for modern drug delivery. The physical properties and non-toxicity of chitosan and chitosan derivatives make it an ideal material for the creation of chitosan-based nanomaterials and their use in nanomedicine especially in oncological treatment. The special focus of the studies carried out so far has been on the development of drugs against tumor cells.

The requirements of chitosan for its use in nanomedicine—drug formulations provide many new solutions and applications in the development of modern medicine. The use of chitosan for the construction of nanoparticles is very important in this case. Chitosan-based nanoparticles can be used for the delivery of active ingredients, such as drugs or natural products, by diverse routes of administration such as oral and parenteral delivery. Nowadays chitosan nanoparticles have become of great interest for nanomedicine, biomedical engineering and the development of new therapeutic drug release systems. They improved bioavailability, increased specificity and sensitivity, and reduced pharmacological toxicity of studied drugs.

Currently, cancer disorders are one of the most important global problems. Our review provides the most important information on the effectiveness of nanomedicine in oncological treatment. The scientific studies give special attention to recent advances in chitosan nano-delivery for cancer treatment. The combinations of chitosan-based nanomaterials with such oncological drugs as doxorubicin, paclitaxel, rapamycin, lactoferrin, tamoxifen, docetaxel, letrozole, gefitinib and 5-fluorouracil, were studied. The researches reveal good outcomes. The use of chitosan nanomaterials in drugs used in oncological treatment have shown enhancement of drug delivery to tumours and improving the cytotoxicity effect on cancer cell lines.

Additionally, studies on the use of chitosan-based nanomaterials in combination with plant-derived secondary metabolites like curcumin, silibinin and polyphenols also have provided promising results. Moreover, the application of chitosan-based nanomaterials in the discovery and development of e.g. antibacterial, anti-inflammatory, antidepressant and antihypertensive formulations which could be used in the treatment of other diseases, was tested. The performed studies have revealed that chitosan-based nanomaterials showed significant enhancement of drug bioavailability drug loading efficiency, drug-releasing capacity and drug encapsulation efficiency. The latest advantages of chitosan nanoparticles applications in nanomedicine are supported also by pre-clinical and clinical studies.

## Data Availability

Not applicable.
